# Triple Infections With Campylobacter coli, Cytomegalovirus, and SARS-CoV-2 in a Lymphoma Patient Treated With Epcoritamab

**DOI:** 10.7759/cureus.103323

**Published:** 2026-02-09

**Authors:** Osamu Imataki, Hina Takagi, Tomoya Ishida, Makiko Uemura

**Affiliations:** 1 Hematology, Kagawa University, Miki, JPN; 2 Hematology, Kagawa University Hospital, Miki, JPN

**Keywords:** campylobacter coli, covid-19, cytomegalovirus, epcoritamab, immunocompromised hosts, malignant lymphoma, sars-cov-2

## Abstract

Malignant lymphoma is a common hematological malignancy, characterized by immunocompromise due to lymphocytic dysfunction. Chemoimmunotherapy for malignant lymphoma, including corticosteroids, rituximab (R), and Bruton's tyrosine kinase inhibitors, depletes normal lymphocytes and disrupts lymphocyte function. Muchmore, various novel treatment modalities, including antibody-drug conjugates, chimeric antigen receptor T cell (CAR-T) therapy, and bispecific T-cell engager (BiTE) antibody treatments such as epcoritamab, are all utilized to target the patient’s own B lymphocytes targeted via mature B cell molecules. The latest anti-lymphoma therapy, epcoritamab, can promote CD3+ T lymphocytes to attack CD20+ normal B lymphocytes and lymphoma cells. Consequently, heavily treated lymphoma patients may experience compromised lymphocyte function. We treated a case of relapsed malignant lymphoma, infected with triple infections, Campylobacter coli, Cytomegalovirus, and SARS-CoV-2, during treatment with epcoritamab after standard chemoimmunotherapies, including R-cyclophosphamide, doxorubicin, vincristine, and prednisolone (CHOP), and R-bendamustine. After three cycles of epcoritamab therapy, the patient developed moderate COVID-19 pneumonia requiring oxygen therapy. Concurrently, he had a bloodstream infection with Campylobacter coli due to Campylobacter coli enterocolitis and Cytomegalovirus antigenemia. His treatment for infections included remdesivir, meropenem, and ganciclovir. By day 12, his infectious diseases improved, and he was discharged in complete recovery. However, he had persistent SARS-CoV-2 viral shedding for six weeks or longer. Epcoritamab can demonstrate long-standing B-cell depletion; however, a long-term influence on T cells is still elusive. This case suggested that we should pay special attention to patients with B-cell manipulating therapy including R, CAR-T, and BiTEs such as epcoritamab.

## Introduction

Malignant lymphoma is one of the most common hematological malignancies characterized by immunocompromise due to lymphocytic dysfunction. Chemoimmunotherapy for malignant lymphoma, including corticosteroids, rituximab, and Bruton's tyrosine kinase inhibitors, depletes normal lymphocytes and predicts poor protection against SARS-CoV-2 [1,2]. Consequently, lymphoma patients are vulnerable to various viral infections, including SARS-CoV-2 [1]. Muchmore, anti-CD20 treatments, such as rituximab therapy, enhance the incidence, ICU admission, and mortality for COVID-19 [2].

During endemic periods, patients treated with rituximab should exercise significant caution in contacting personnel and avoiding crowded places. Lymphoma patients receiving B-cell-depleting therapies are known to have markedly impaired vaccine responses [3]. Pharmacological prophylaxis such as tixagevimab/cilgavimab (Evusheld®) has been an important preventive strategy in many countries during the COVID-19 endemic period [4]. Not only SARS-CoV-2, in consequence lymphoma patients are vulnerable to bacterial and fungal pathogens due to disease-related and treatment-related immunosuppression [5]. Campylobacter coli can cause severe and sometimes systemic infections in immunocompromised hosts [6]. Cytomegalovirus (CMV) reactivation is a well-recognized complication in patients with hematologic malignancies [7].

It is widely known that cancer patients, including those with hematological malignancies, have an increased risk of contracting COVID-19 and experiencing poor prognosis [8]. Furthermore, long COVID-19 syndrome has been reported as a specific condition among vulnerable cases, such as those undergoing treatment for hematological malignancies [9]. The phenomenon of prolonged COVID-19 infection with persistent shedding of SARS-CoV-2 RNA is now widely accepted [9]. In a case series reported by Faxén et al., the authors documented persistent SARS-CoV-2 detection in four lymphoma patients [10]. The management of symptoms associated with long-standing COVID-19 has been specifically addressed by the National Institute for Health and Care Excellence (NICE) in their statement on long COVID-19 research [11].

B-cell depletion and altered T-cell dynamics might predispose to multi-pathogen infections including COVID-19 [12]. B-cell depletion not only impairs humoral immunity and vaccine responsiveness but also alters T-cell dynamics, thereby creating a state of combined immune vulnerability. This immunologic milieu predisposes patients to multi-pathogen infections, including bacterial, viral, and opportunistic pathogens. Targeting mature B cells expressing CD19, CD20, or CD22 is a well-established standard for the treatment of B-cell malignant lymphoma. Building on this concept, novel treatment modalities have been developed, including antibody-drug conjugates, chimeric antigen receptor T cell (CAR-T) therapy [13,14], and bispecific T-cell engager (BiTE) antibody treatments such as blinatumomab [15] and epcoritamab [14,16]. Additionally, the latest anti-lymphoma therapy, epcoritamab [17], can promote CD3+ T lymphocytes to attack CD20+ normal B lymphocytes and lymphoma cells. Consequently, heavily treated lymphoma patients may experience compromised lymphocyte function. BiTEs such as epcoritamab induce continuous T-cell engagement and activation, which may predispose to T-cell functional exhaustion [18]. Infection risk is recognized in patients receiving B-cell-depleting therapies; there is very limited evidence regarding the concurrent bacterial and viral infections in those treated with BiTEs such as epcoritamab. This case illustrates the rarity and clinical significance of a triple infection involving both bacterial (Campylobacter coli) and viral (SARS-CoV-2 and CMV) pathogens under epcoritamab therapy. It highlights the importance of comprehensive infection surveillance and preventive strategies in this population.

## Case presentation

We treated a 78-year-old man with relapsed follicular lymphoma. The patient had previously been treated with cyclophosphamide, doxorubicin, vincristine, prednisolone, and rituximab (R-CHOP), followed by R-bendamustine for relapse, and finally epcoritamab as third-line treatment. The patient’s comorbidities were hypertension and diabetes mellitus. The patient does not have a prior infection history. A vaccination for SARS-CoV-2 was completed four times two years ago, and no additional vaccinations were performed. ECOG performance status was 1, and there were no baseline organ dysfunctions.

**Figure 1:**
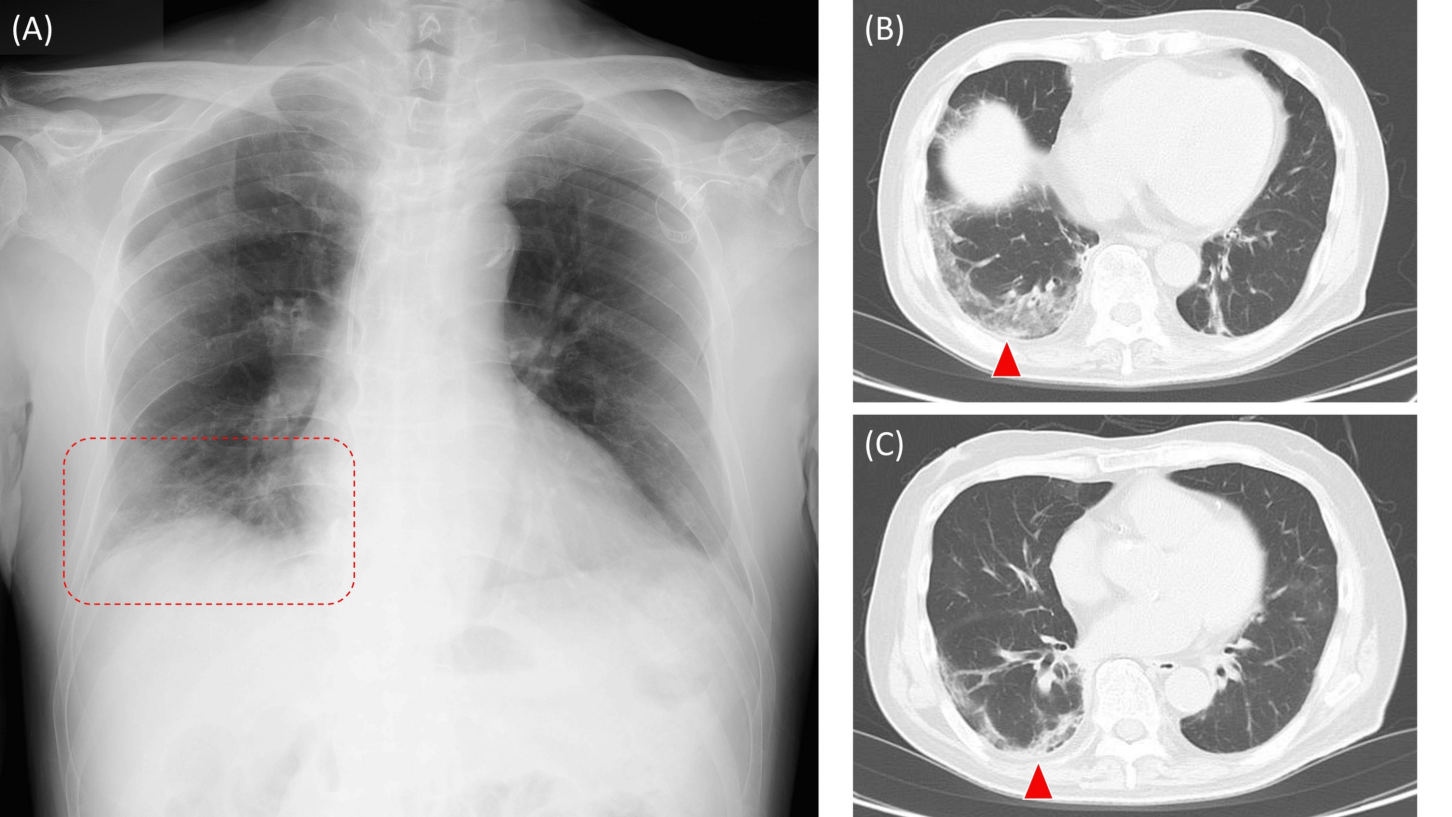
The patient’s chest X-ray and CT scanning (A) Dense consolidation was observed in the right lower lung field on the chest X-ray (red dotted area). Ground glass opacity spread peripherally along the dorsal pleural side in the right lung in the chest CT imaging (window level -500HU, window width 1200HU, 5mm slice) at the right diaphragm level (B) and left atrial level (C) (red arrowheads).

**Figure 2:**
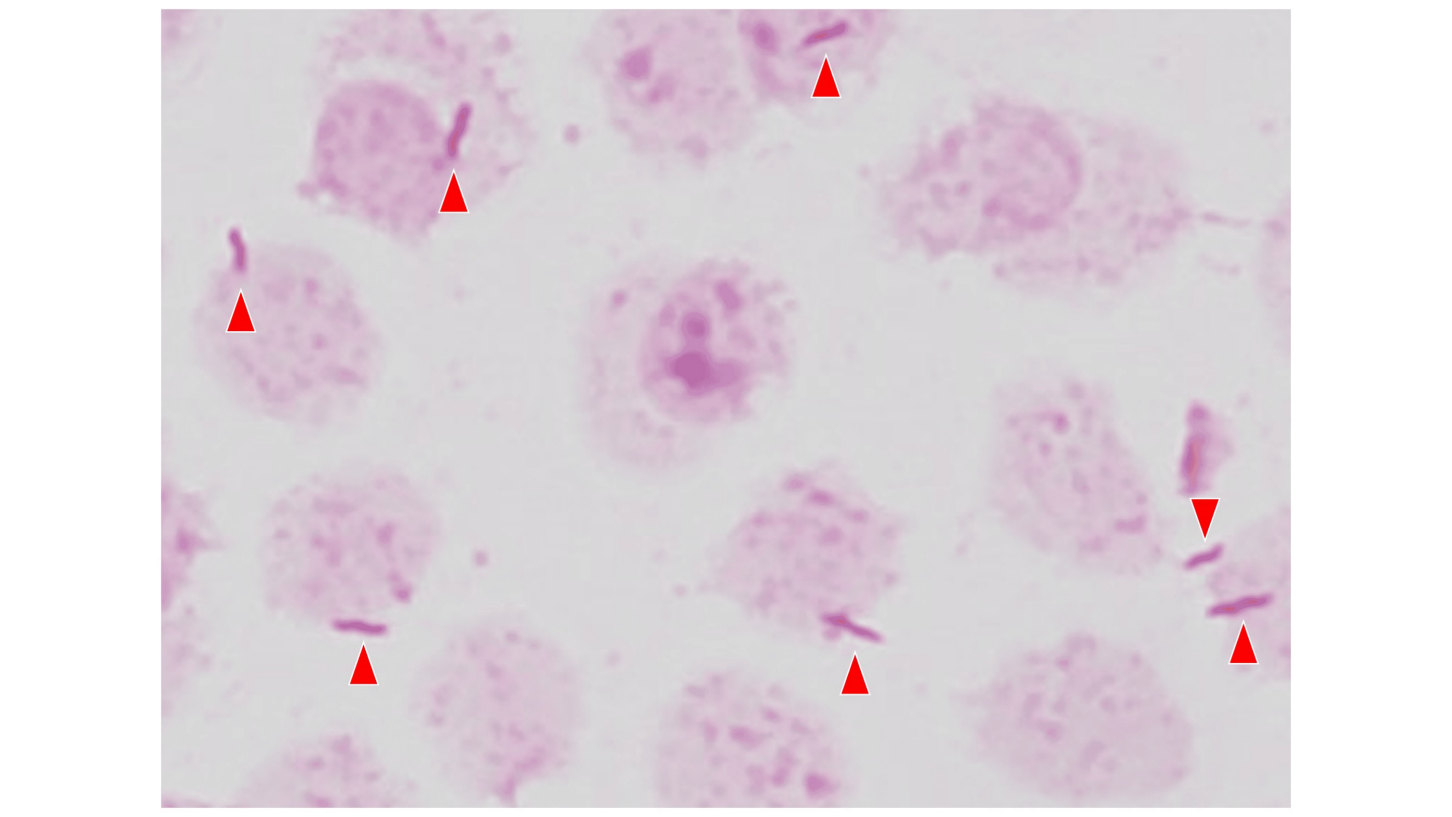
Gram staining of culture bottle obtained from the patient’s blood Gram-negative spiral bacteria forming “gul-wing” have grown (red arrowheads) in the aerobic culture bottle. Campylobacter species typically appear as gull‑wing pairs, S‑shaped with a short spiral (1–2 turns) curve. This microorganism was identified as Campylobacter coli by matrix-assisted laser desorption ionization/time of flight (MALDI-TOF-MAS) and traditional culture assays.

The patient received epcoritamab therapy as the following doses: 0.16 mg at day 1, 0.8 mg at day 8, and 48 mg at day 15 and 22, for cycle 1; 48 mg weekly at day 1, 8, 15, and 22 for cycles 2 and 3. The patient's course did not require tocilizumab therapy for the adverse events such as cytokine release syndrome or tumor flare reaction. On day 29 of cycle 3 of epcoritamab therapy, the patient developed SARS-CoV-2 infection with COVID-19 pneumonia (moderate, requiring oxygen therapy). The patient’s chest X-ray and CT scan demonstrated right-sided peripheral ground-glass opacities suggestive of viral pneumonia (Figure 1). At the onset of COVID-19, the patient also had a Campylobacter coli bloodstream infection (gram-negative spiral bacteria in culture bottle) (Figure 2) accompanied by Campylobacter coli enterocolitis (symptomatic positive stool culture), and CMV antigenemia (positive in 2 and 3 cell counts, pp65 (C10/11) indirect enzyme immunoassay, threshold <1) on the day 29 of cycle 3. Streptococcus pneumoniae, Legionella species, and influenza viruses A and B were negative by the assay. No microorganisms were grown in the culture of the patient’s sputum. The patient consumed a raw egg one week before the onset of his infectious disease. The patient was not prescribed any antimicrobials for prophylaxis.

**Figure 3:**
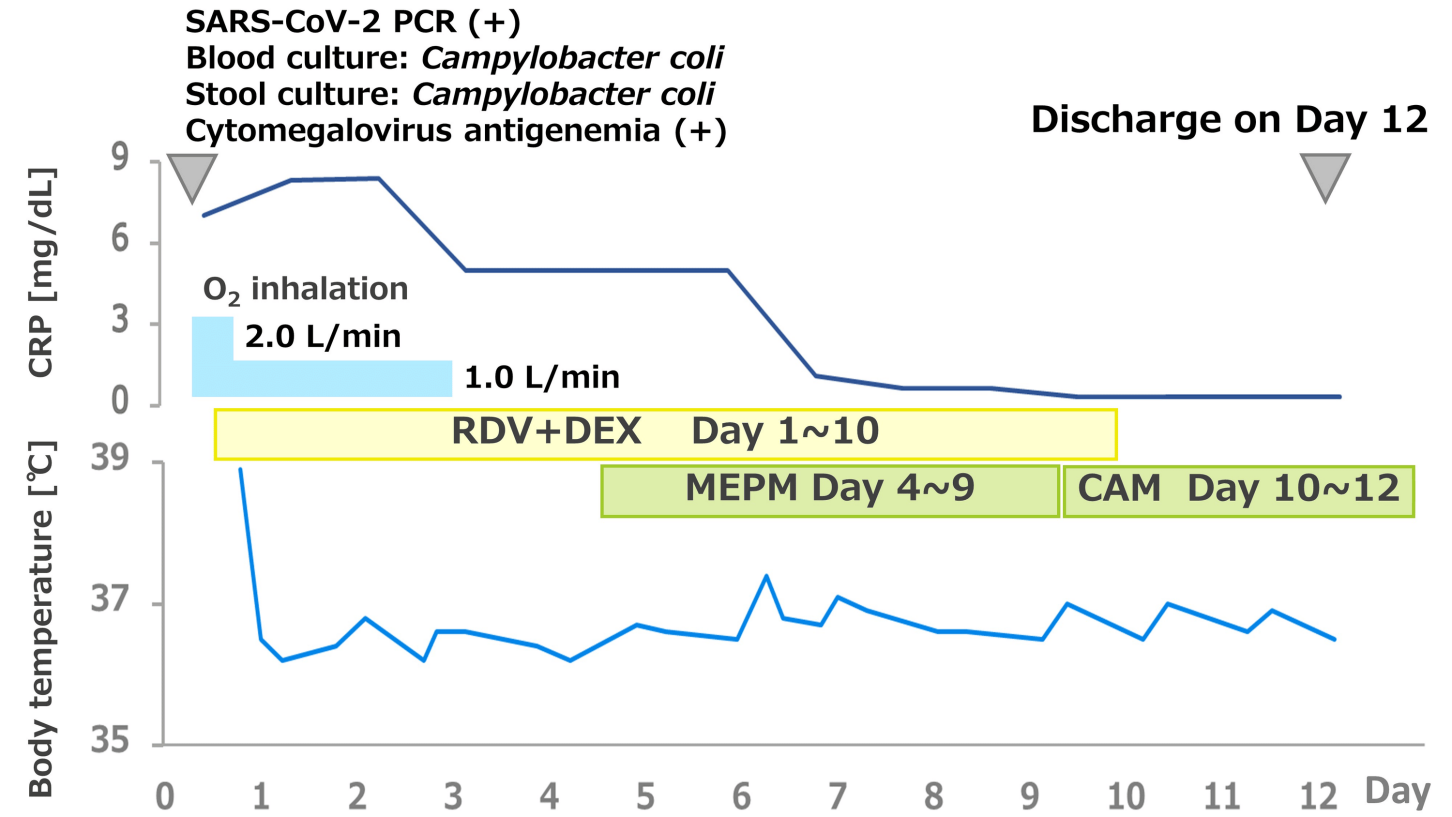
The clinical course of the patient Initially, the patient was administered remdesivir (RDV) at a dose of 200 mg/day on the initial day followed by 100 mg/day for a total of 10 days and dexamethasone (DEX) 6.6 mg/day for seven days. Oxygen inhalation was required for the first three days after hospitalization. Meropenem (MEPM) at a dose of 1.0 g every eight hours was added to COVID-19 therapy on day 4, according to the results of blood and stool cultures at the onset. The patient’s right lower lung field consolidation on the chest X-ray was cleared on day 6. The second blood culture was negative on day 8 with a clearance of the patient’s pyrexia and respiratory symptoms. MEPM was discontinued after seven days of treatment, and clarithromycin (CAM) 400 mg/day was administered after discharge, based on the drug susceptibility of the causative pathogen. The patient was given 450 mg/day of valganciclovir to treat cytomegalovirus antigenemia throughout their hospitalization.

The patient’s laboratory data are indicated in Table 1. The treatment course of the patient is indicated in Figure 3. The patient's lymphocytes were 2091/μL, CD3+ T cells were 1503/μL (71.9%, reference 58.0~84.0%), and CD19+ B cells were 2/μL (0.1%, reference 3.5~15.0%). A lymphocyte subset was CD4+/CD8+ 0.24 (13.3%/54.5%) (reference ratio 0.40~2.30), and IgG was 516 mg/dL (reference 861~1747). The patient was treated with remdesivir (200 mg/day on day 1 followed by 100 mg/day for nine days), meropenem (1.0 mg three times daily for six days), and ganciclovir (5 mg/kg/day, three times a week) for the triple infections, COVID-19, Campylobacter coli enterocolitis, and Cytomegalovirus antigenemia, respectively. The patient’s infectious diseases showed improvement by day 12. The patient’s chest X-ray and chest CT were followed up on. The patient was discharged in clinical recovery, capable of performing daily activities. The patient’s chest CT was followed up three weeks later and the pulmonary consolidative lesion had disappeared. The patient had persistent SARS-CoV-2 viral shedding for six weeks or longer [19,20]. The quantitative SARS-CoV-2 antigen assay was performed even after the patient’s discharge, and it decreased gradually to 5000.00 pg/mL (reference value <0.99) on day 22, 180.11 pg/mL on day 28, 213.33 pg/mL on day 35, and <0.60 pg/mL on day 56.

**Table 1:** Patient’s laboratory data MCV: Mean corpuscular volume; PLT: platelet; HCT: hematocrit; BUN: blood urea nitrogen; AST: aspartate aminotransferase; ALT: alanine transaminase; FIB: fibrinogen blood test ; FDP: fibrin degradation product

Count of blood cells			
Parameter	Value	Unit	Reference range
WBC	6,970	/μL	3,300-8,600
Stab	1.0	%	0.5-6.5
Seg	66.0	%	38-74
Mono	2.5	%	2-10
Lymph	30.0	%	16.5-49.5
Eosino	0.5	%	0-8.5
Baso	0.0	%	0-2.5
RBC	294×10^4^	/μL	435-555
HGB	9.2	g/dL	13.7-16.8
HCT	28.0	%	40.7-50.1
MCV	95.2	fL	83.6-98.2
RET	40,900	/μL	30,000-90,000
PLT	14.1×10^4^	/μL	15.8-34.8
Biochemistry			
Parameter	Value	Unit	Reference range
CRP	8.32	mg/dL	0-0.14
TP	4.8	g/dL	6.6-8.1
ALB	2.8	g/dL	4.1-5.1
BUN	14.8	mg/dL	8-20
CRE	0.94	mg/dL	0.65-1.07
UA	4.9L	mg/dL	3.7-7.8
T-BIL	0.5	mg/dL	0.4-1.5
D-BIL	0.1	mg/dL	0-0.3
AST	19	U/L	13-30
ALT	18	U/L	10-42
LDH	422	U/L	124-222
γGTP	88	U/L	13-64
CHE	193	U/L	240-486
Na	137	mmol/L	138-145
K	3.8	mmol/L	3.6-4.8
Cl	101	mmol/L	101-108
Ca	7.8	mg/dL	8.8-10.1
IP	2.4	mg/dL	2.7-4.6
Mg	1.6	mg/dL	1.8-4.6
CPK	34	U/L	59-248
SAMY	47	U/L	44-132
BS	94	mg/dL	73-109
Coagulofibrinolysis			
Parameter	Value	Unit	Reference range
PT-INR	0.89		-
APTT	28.0	Sec	24-34
FIB	452	mg/dL	200-400
AT	85.0	%	80-130
FDP	3.6	μg/mL	0-5
D-dimer	1.8	μg/mL	0-1

The patient did not receive tixagevimab/cilgavimab prophylaxis prior to SARS-CoV-2 infection. We also clarified the reason at the time of presentation, Evusheld® was not available at our institution. In Japan, Evusheld® was approved by the drug organization in September 2022 and broadly prescribed up to August 2023 in Japan, as well our institute [4]. The second shot of Evusheld® was not generalized in Japan because of the cost and eligibility since September 2023.

## Discussion

Campylobacter infections predominantly affect the elderly and immunocompromised individuals. Immunosuppressed patients are often prone to developing Campylobacter bloodstream infections. The treatment for enteral Campylobacter infections includes erythromycin, ciprofloxacin, or doxycycline. For invasive infections, broader-spectrum antibiotics such as meropenem are optimal. The most common gram-negative spiral pathogen causing bacteremia affected to immunocompromised individuals is Helicobacter cinaedi [21]. As shown in our case, Campylobacter spp. including Campylobacter fetus, is also a leading spiral microorganism causing bacteremia in vulnerable patients [22,23]. While Campylobacter coli is typically associated with gastrointestinal disease and opportunistic infections in immunocompromised hosts, other species, such as Campylobacter fetus, cause severe invasive disease even in immunocompetent individuals. Helicobacter spp. and Campylobacter spp. are the most leading spiral pathogens affecting immunocompromised patients. The infection caused by these pathogens is opportunistic in immunocompetent patients.

These two organisms share the following microbiological properties: (i) They form the spiral-shaped morphology, Gram-negative bacilli. (ii) They reside in the host as reservoir microbes found in the gastrointestinal tract of humans and animals. They can be transmitted from the gut to the systemic circulation and are likely to translocate from the gut, especially in immunocompromised hosts. (iii) These organisms commonly cause disease in specific risk groups, including patients with HIV, hematologic malignancies, those undergoing immunosuppressive therapy, and the elderly. As a clinical manifestation of these opportunistic infections, bacteremia (often recurrent), cellulitis, and undetected fever are observed. In rare cases, infected aneurysms are a critical or lethal complication of these opportunistic organisms [24,25]. In addition to these shared properties that cause bloodstream infections in immunocompromised hosts, we can explain case-specific presentations, such as mucosal barrier injury and impaired bacterial clearance due to neutropenia or lymphopenia. Reduced T-cell surveillance has also been suggested as a risk factor for these intracellular pathogens [26]. Given these points, we must monitor patients with these opportunistic bacteria to prevent severe illness.

Epcoritamab, a bispecific antibody targeting CD20 on B cells and CD3 on T cells, is designed to activate rather than suppress T cells. BiTEs including epcoritamab have been reported to raise concerns about increased infection risk [27]. This review article systematically reviewed opportunistic infections in patients with malignant lymphoma treated with bispecific antibodies, including epcoritamab. It included data from multiple clinical trials and observational cohorts. Reported infections included CMV reactivation, Pneumocystis jirovecii pneumonia, Herpes zoster, and various bacterial sepsis. Some risk factors have been identified for these opportunistic infections. They reported that fatal infections occurred in an incidence of 3%. However, there are no guidelines for anti-infective prophylaxis and preventive procedures. BiTEs, including epcoritamab, should now be listed as a risk agent for opportunistic infections. The review emphasizes the need for infection surveillance and prophylaxis during and after therapy. In our case, the potential confounding effects of prior immunochemotherapy and advanced age can contribute to the infectious episodes. In either situation, a specific prophylaxis regimen such as acyclovir/ganciclovir and fluoroquinolones could help achieve a better outcome during BiTE therapy.

This case indicates that immunological surveillance and mucosal protection against infectious pathogens, including bacteria and viruses, by T cells might be compromised by multiple lines of therapy, including epcoritamab. In vitro studies have shown that epcoritamab can activate terminally differentiated exhausted T cells to target and kill CD20-expressing B cells. Additionally, clinical trials have reported robust and sustained B-cell depletion, along with T-cell activation and expansion [28]. Our suggestion of T-cell dysfunction or exhaustion is speculative, as no functional assays or longitudinal immune profiling were performed. T-cell exhaustion is a possible explanation for the observed clinical course, while emphasizing that this remains hypothetical and requires further validation in future studies. Epcoritamab can demonstrate long-standing B-cell depletion. However, it remains unclear whether epcoritamab ultimately exhausts T cells after prolonged activation. Cytokine release after epcoritamab therapy can impair and dysregulate homeostatic immune functions [29]. Therefore, we should monitor opportunistic infections in immunocompromised patients long after they undergo chemotherapy, including treatment with epcoritamab. We anticipate that patients treated with epcoritamab may be susceptible to certain microorganisms. The BiTEs are now widely accepted for treating solid malignancies [30,31]. Clinical oncologists can refer to the impact of similar immunosuppression in their patients, managing it accordingly.

## Conclusions

Epcoritamab can cause B-cell depletion, cytokine release, and T-cell activation. In our speculation, these effects may compromise immune surveillance and increase the risk of infection in immunocompromised patients. We hope that our experience will support treating odd opportunistic infections, including those caused by multiple microorganisms, after epcoritamab. Furthermore, we will gather and analyze comprehensive infectious disease data associated with BiTE therapy based on clinical data and pharmacovigilance reports. In conclusion, this case treated with epcoritamab highlights profound immunocompromise during epcoritamab-induced B‑cell depletion and possible T-cell exhaustion in relapsed lymphoma.
